# Antibacterial and Antibiofilm Activities of Novel Cyclic Peptides against Methicillin-Resistant *Staphylococcus aureus*

**DOI:** 10.3390/ijms23148029

**Published:** 2022-07-21

**Authors:** Guoxing Wei, Yun He

**Affiliations:** Chongqing Key Laboratory of Natural Product Synthesis and Drug Research, School of Pharmaceutical Sciences, Chongqing University, Chongqing 401331, China

**Keywords:** MRSA infections, antibiotic tolerance, cyclic peptide, antibiofilm, persister

## Abstract

Methicillin-resistant *Staphylococcus aureus* (MRSA) has led to serious infections, especially in hospitals and clinics, where treatment and prevention have become more difficult due to the formation of biofilms. Owing to biofilm-derived antibiotic tolerance, the currently available traditional antibiotics have failed to treat MRSA infections. Hence, there is a urgent need to develop novel antibiotics for treating life-threatening MRSA infections. Lugdunin (cyclic peptide-1), a nonribosomal cyclic peptide produced by *Staphylococcus lugdunensis*, exhibits potent antimicrobial activity against MRSA. Amazingly, cyclic peptide-1 and its analogues cyclic peptide-11 and cyclic peptide-14 have the ability to disperse mature MRSA biofilms and show anti-clinical MRSA activity, including MRSA persister cells. In addition, these three cyclic peptide compounds have non-toxicity, lower hemolytic activity and lack of resistance development. Our results indicate that cyclic peptide-1, cyclic peptide-11, and cyclic peptide-14 have great potential as new antimicrobial drug candidates for the treatment of clinical MRSA infections.

## 1. Introduction

With the popular use of antibiotics, most antibiotics are ineffective against multidrug-resistant bacteria, so patients with associated infections have few available treatment options [1]. Resistant bacteria, especially the emergence of multidrug-resistant bacteria, have become a threat to public health [2,3]. WHO warns that multidrug-resistant bacterial infections will surpass cancers as the largest threat to human health if no effective actions are taken in the coming decades [4]. Multiple factors can lead to the emergence of multidrug-resistant bacteria, such as the formation of bacterial biofilms, which are responsible for 60–80% of chronic infections [5,6,7]. Bacterial biofilms can be defined as a cluster of bacteria and self-produced extracellular polymeric substances, such as polysaccharides, lipids extracellular DNA, and proteins [8]. Bacteria living in their biofilm-mode of growth contribute greatly to the emergence of drug resistance and have demonstrated an up to 1000-fold reduced susceptibility to most antibiotics. A biofilm can prevent the interaction between antibiotics and bacterial cells and protect bacteria from forming various environmental stresses [9,10,11,12], and host immune system biofilm-associated infections are extremely difficult to treat with conventional antibiotics, causing chronic infections and nonhealing wounds, such as endocarditis, cysticfibrosis, and soft tissue infections caused by MRSA [13,14,15,16]. In addition, persister bacteria are dormant, super-antibiotic-resistant cells that can reside in a biofilm state within or external to mammalian cells, so antibiotics fail to eradicate them [17,18]. Bacterial biofilms have several potential antimicrobial resistance mechanisms, mainly including (i) a biofilm extracellular matrix that blocks the penetration of antibiotics, (ii) biofilm components that sequester antibiotics, and (iii) an inner environment that lacks nutrients and oxygen, which can cause bacteria to become metabolically inactive and thus survive a low dose of antibiotics [19,20,21]. However, most conventional antibiotics are designed for drug targets such as DNA replication, cell wall biogenesis, and a protein synthesis of bacteria growing in a planktonic state. These processes are hard to be effective in the biofilm-grown bacterial population. Hence, novel therapies addressing bacterial infections must take the presence of biofilms into consideration [22,23].

In order to overcome the biofilm extracellular matrix obstacle, compounds need to be specifically designed to inhibit the biofilm formation and dispersion of mature biofilms. Anti-biofilms strategies involving bacteriophages and polymers are currently being investigated [24]. In response to this obstacle, attention has been drawn to the development of antimicrobial peptides (AMPs) as a novel class of antibiotics [25,26]. AMPs that play important roles in the innate human immune system have attracted special attention as an alternative to conventional antibiotics to prevent and treat bacterial infections, especially for treating biofilm-associated infectious diseases [27,28,29,30], owing to their broad spectrum activity, low incidence of resistance development, and ability to kill stationary phase “persister” cells through a membrane-active mode of action [31]. Confronting increasingly serious drug-resistant bacteria infections, especially biofilm infections and persister bacteria infections, AMPs are considered to be the most ideal solution.

Cyclic peptide-1 is a nonribosomal cyclic peptide produced by *Staphylococcus lugdunensis* and features a thiazolidine ring as part of the backbone, showing potent antimicrobial activity against MRSA and excellent bactericidal effects on the skin of mice infected with *S. aureus* [32]. In addition, the essential motifs for antimicrobial activity have also been identified in structure–activity relationship studies of cyclic peptide-1 [33]. Therefore, in this work, we synthesized cyclic peptide-1 and analogues cyclic peptide-11 and cyclic peptide-14 by structure–activity relationships and assessed their antibacterial and antibiofilm activities of MRSA in vitro. Cyclic peptide-1 and these analogues may develop promising and novel antimicrobials to deal with increasingly serious MRSA infections, including biofilm infections and persister bacteria infections.

## 2. Results

### 2.1. Antibacterial Assessment of Cyclic Peptides

To assess the antibacterial activities of these cyclic peptides against MRSA, activity assays were performed according to standard microbroth dilution methods. The MICs of these peptides were 5.1–10.6 μM, and the MBCs of these peptides were 21.2–84.8 μM (Table 1).

In addition, we also determined their antimicrobial activities against other bacteria, such as *Enterococcus faecium*, *Acinetobacter baumannii*, *Klebsiella pneumoniae*, *Pseudomonas aeruginosa*, and *Enterobacter* sp. (Table 2). However, they had no obvious antibacterial activity, indicating that these peptides have narrow-spectrum antibacterial activities.

### 2.2. Time-Kill Studies against MRSA

**Figure 1:**
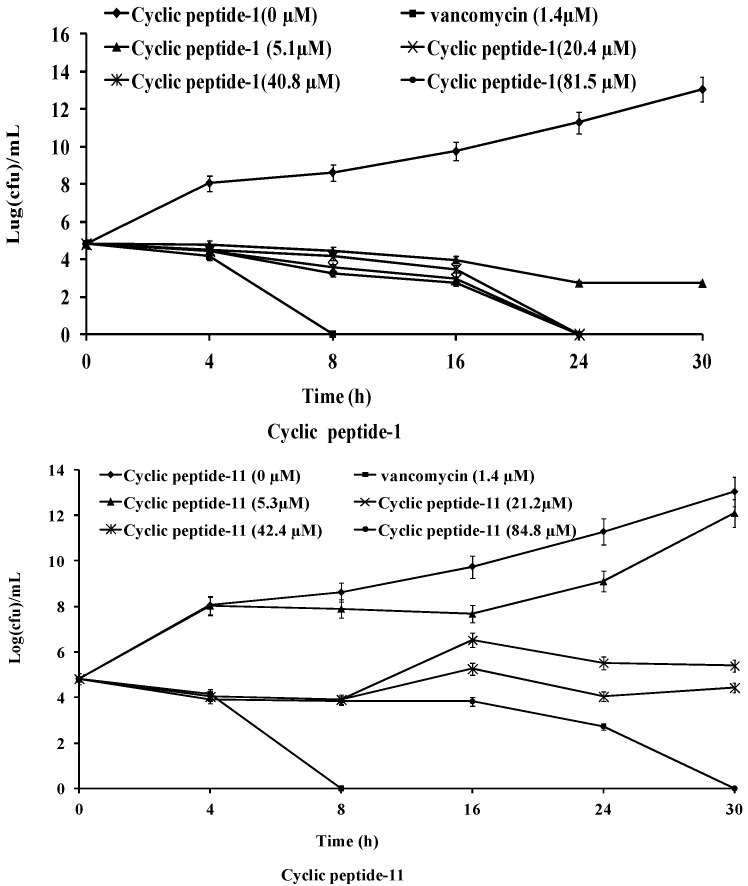

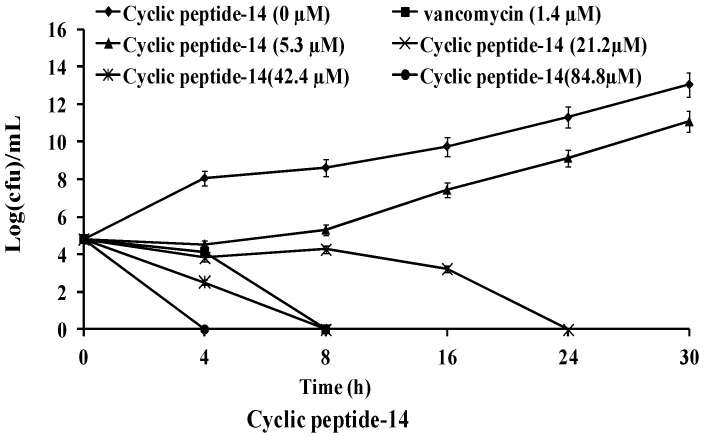
Time-kill curves of cyclic peptide-1, cyclic peptide-11, and cyclic peptide-14.

The killing kinetics of these three cyclic peptide compounds against MRSA ATCC43300 were analyzed by time-kill assays. As shown in Figure 1, MRSA ATCC43300 was completely killed within 24 h of incubation with cyclic peptide-1 at 4 × MIC. However, MRSA ATCC43300 was not completely killed until 30 h at 1 × MIC, leaving a growing litter bacteria. MRSA ATCC43300 was completely killed treating with cyclic peptide-11 within 8 h of incubation at 8 × MIC, while significant regrowth was observed at 1 × MIC, 2 × MIC, and 4 × MIC. Cyclic peptide-14 completely killed MRSA ATCC43300 within 4 h of incubation at 8 × MIC, within 8 h of incubation at 4 × MIC, and within 24 h of incubation at 2 × MIC. In addition, significant regrowth was observed at 1 × MIC after 24 h of incubation, indicating that cyclic peptide-14 is bacteriostatic at 1 × MIC.

### 2.3. The Inhibition and Dispersal of Bacterial Biofilms

**Figure 2:**
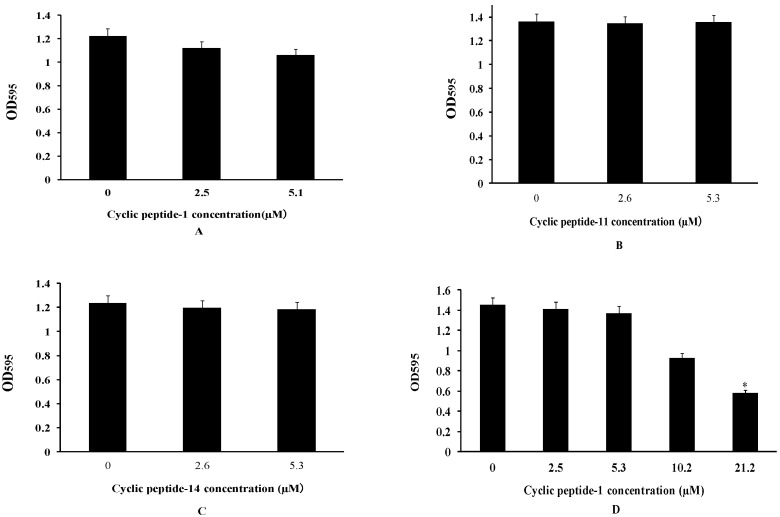

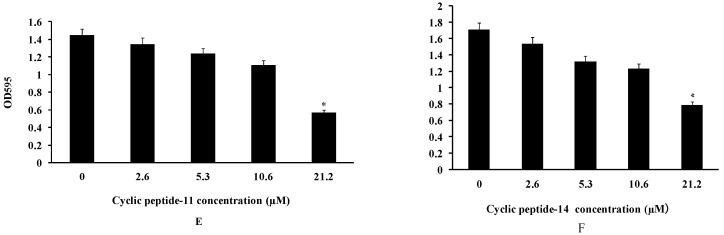
Cyclic peptide-1 (**A**), cyclic peptide-11 (**B**), and cyclic peptide-14 (**C**) prevent biofilm formation. Cyclic peptide-1 (**D**), cyclic peptide-11 (**E**), and cyclic peptide-14 (**F**) disperse established biofilms. A *t* test was used to determine the statistical significance of biofilms treated at 21.2 μM versus untreated conditions (* *p* < 0.05).

To investigate whether these three cyclic peptide compounds can produce positive efficacy of anti-MRSA biofilms, the activities of three cyclic peptide compounds were determined by the inhibition and dispersal of bacterial biofilms assays with MRSA3390. The abilities of cyclic peptides that inhibit the formation of biofilms and disperse the preformed biofilms of MRSA were investigated by a crystal violet staining experiment. The MRSA3390 biofilm prevented formation after exposure to 10.6 μM of cyclic peptides (Figure 2A–C). However, these cyclic peptide compounds had strong bactericidal activity against MRSA at 10.6 μM. These results illustrate that the prevention of biofilm formation at 10.6 μM may be attributable to the apoptosis of bacteria.

Biofilms are composed of extracellular polymer matrices that cause resistance development. Antibacterial agents that penetrate the EPS matrix of biofilms are essential for treating chronic bacterial biofilm infections [34]. To determine whether these compounds can disperse preformed biofilms, the activity of dispersing biofilms was tested in our study. Our results showed that mature MRSA biofilms could be dispersed at 21.2 μM of cyclic peptide-1, -11, and -14 (Figure 2D–F).

**Figure 3:**
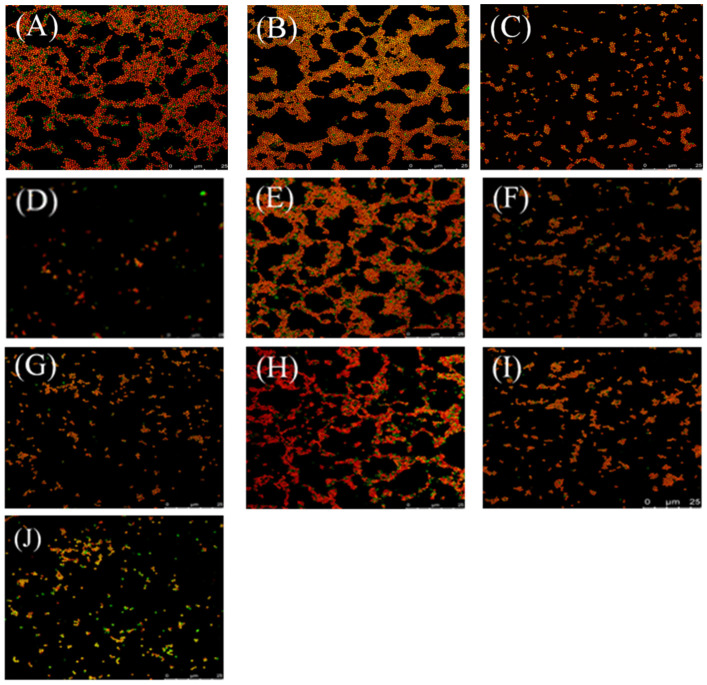
Effect of treating preformed biofilms of MRSA 3390 with cyclic peptide-1, 11, 14 at various concentrations for 24 h. The cyclic peptide-1, 11, 14 were present at 1 × MIC (**B**,**E**,**H**), 5 × MIC (**C**,**F**,**I**) and 10 × MIC (**D**,**G**,**J**) concentrations, respectively. (**A**) Controls with no cyclic peptide added. Biofilm bacteria were stained with SYTO 9 (green: live) and PI (red: dead). Bar = 25 μm.

At the same time, confocal microscopy images of live/dead bacterial stained biofilms were employed to evaluate the efficacy of cyclic peptide compounds against biofilms. As shown in Figure 3, cyclic peptides could disperse preformed biofilms of MRSA3390 at 53 and 106 μM, indicating cyclic peptides could penetrate and disperse the EPS matrix barrier. Therefore, cyclic peptide-1, -11, and -14 may provide a basis for treating biofilm-based infections.

### 2.4. Persister Bacteria

**Figure 4:**
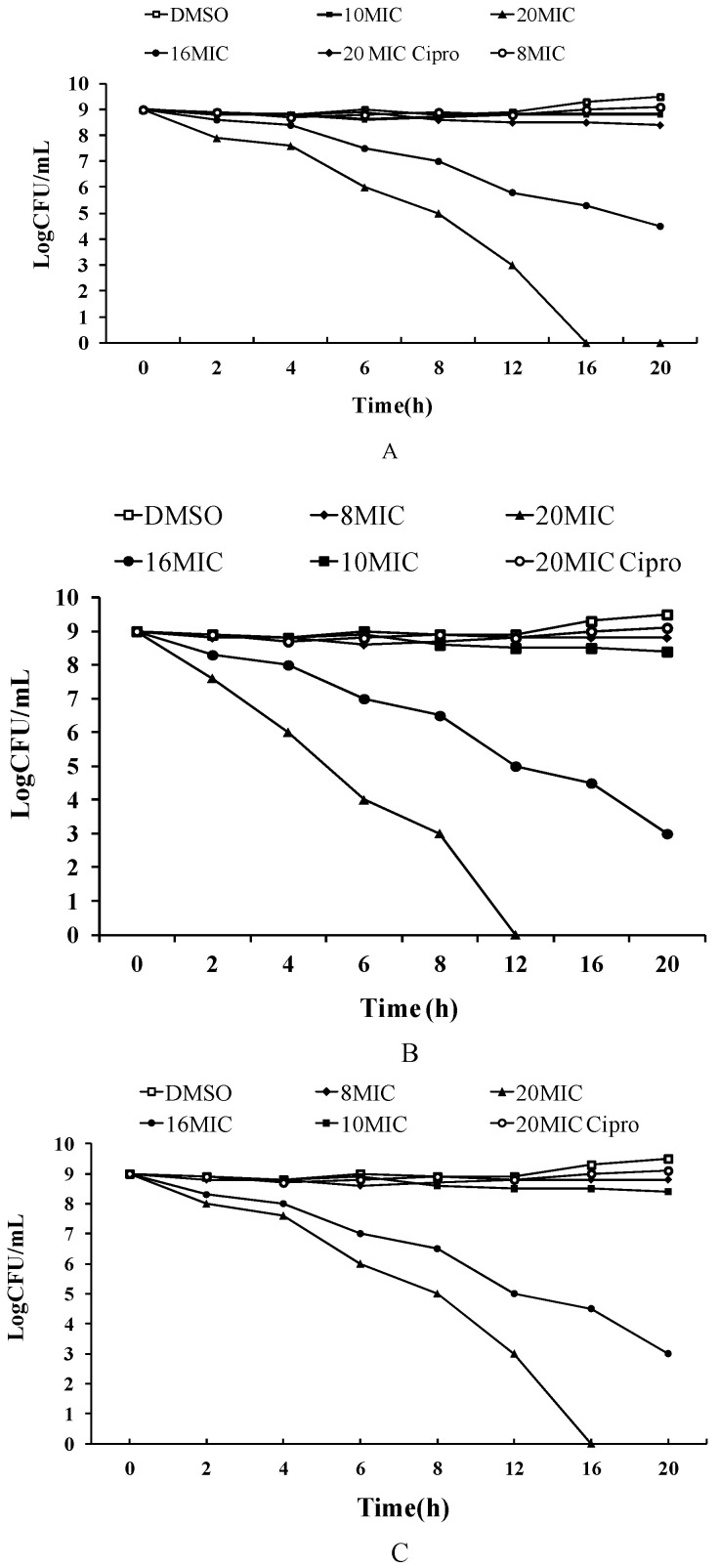
The ability of cyclic peptide-1 (**A**), cyclic peptide-11 (**B**), and cyclic peptide-14 (**C**) to kill MRSA ATCC43300 persisters.

These three cyclic peptide compounds were demonstrated to have a strong anti-MRSA effect and the potential to destroy mature MRSA biofilms. Subsequently, their abilities to kill MRSA ATCC43300 persisters were evaluated (Figure 4). When treated with 16–20 × MIC of cyclic peptides, the numbers of living bacteria decreased sharply over time, and no colony was detected at 20 × MIC after a 16 h treatment of cyclic peptide-1, at 20 × MIC after a 12 h treatment of cyclic peptide-11, or at 20 × MIC after a 16 h treatment of cyclic peptide-14, respectively. Similarly, MRSA ATCC43300 persisters were eradicated when treated with cyclic peptide-11 at 20 × MIC for 16 h, showing its effectiveness in killing MRSA ATCC43300 persisters. This result suggested that these three cyclic peptide compounds are efficient in killing MRSA ATCC43300 persisters.

### 2.5. Resistance Development

**Figure 5:**
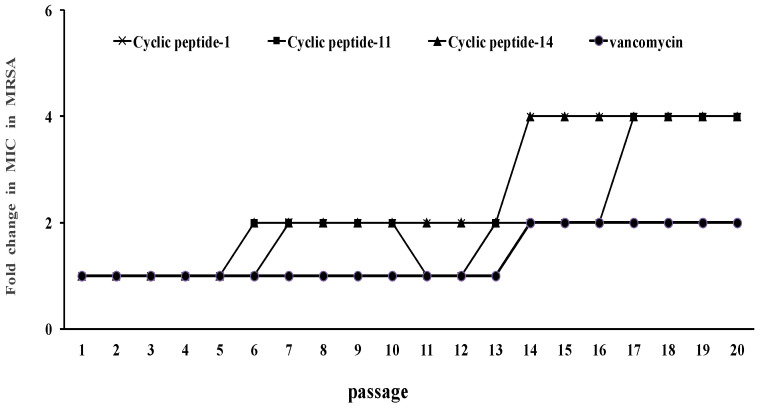
Resistance development of cyclic peptide-1, cyclic peptide-11, and cyclic peptide-14.

The development of resistance to cyclic peptide-1 was assessed as described in previous research [33]. For comparison, the development of resistance to the clinically relevant antibiotic vancomycin was determined. If this antibacterial compound will be developed as an antimicrobial drug, resistance development is a major concern. We measured the ability of MRSA ATCC43300 to develop resistance to cyclic peptide-1, -11, and -14. MRSA ATCC43300 with serial passaging in the subinhibitory concentrations of cyclic peptide-1, -11, and -14 over 20 days did not show any development of resistance to cyclic peptides (Figure 5).

### 2.6. Safety Assessment

**Figure 6:**
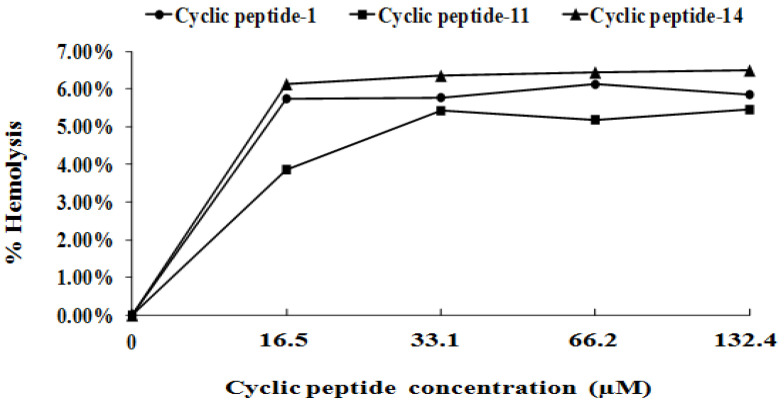
Percentage of hemolysis of rabbit blood cells at various cyclic peptide concentrations.

Minimal hemolytic concentration was determined as the lowest concentration of antimicrobials, which caused 10% hemolysis of erythrocytes. The percentage of hemolysis of cyclic peptide-1, cyclic peptide-11, and cyclic peptide-14 at 132.4 μM was 5.85%, 5.45%, and 6.49% respectively (Figure 6). Our results indicate that cyclic peptide-1, -11, and -14 yield the best therapeutic index.

**Figure 7:**
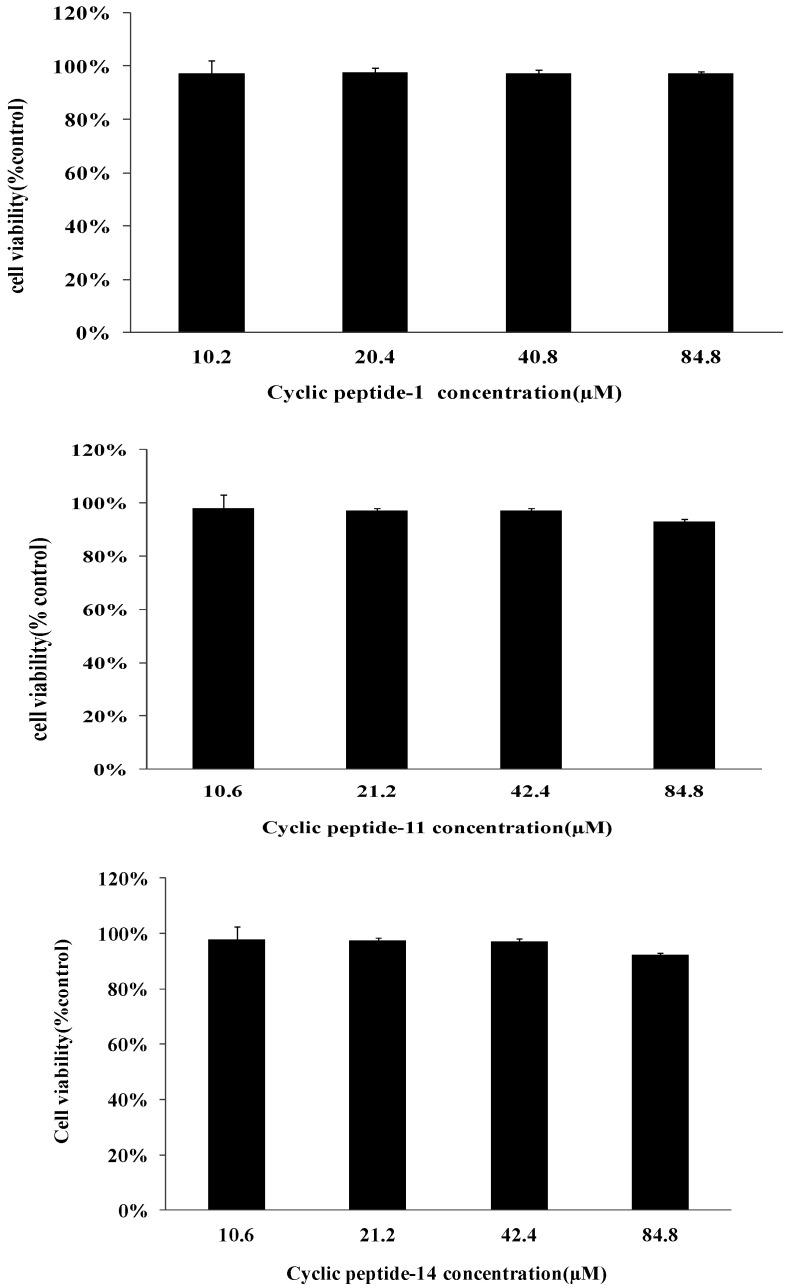
Cytotoxicity assay of cyclic peptide-1, -11, and -14 against human liver L02.

Cell survival after 48 h of incubation with different concentrations of cyclic peptides was evaluated by an MTT assay. The toxicity of cyclic peptide-1, -11, and -14 toward human liver L02 cells is shown in Figure 7. As negative controls, 5‰ DMSO was used. The low toxicity of cyclic peptide-1, -11, and -14 was observed against human liver L02 cells. The high concentrations required for cyclic peptide-1, -11, and -14 (84.8 μM) showed no significant loss in cell viability. These results indicate that cyclic peptide-11 and cyclic peptide-14 are safe for mammalian cells as an antibacterial agent.

### 2.7. Membrane Permeabilization Studies

**Figure 8:**
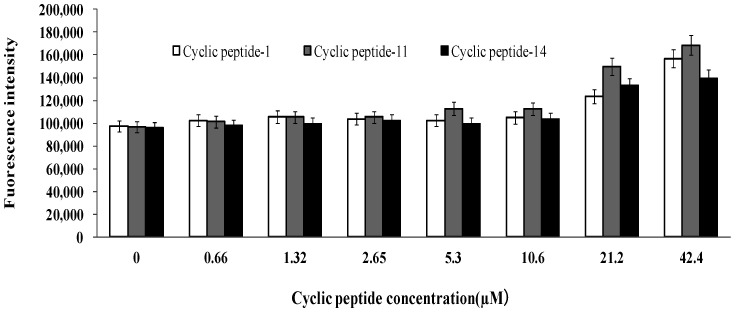
Influence of cyclic peptides on membrane and cellular integrity.

We treated MRSA ATCC43300 with cyclic peptide-1, -11, and -14 and subsequently added propidium iodide, an indicator of membrane and cellular integrity. We found that concentrations of cyclic peptide-1, -11, and -14 less than 10.6 μM had no effect on membrane and cellular integrity (Figure 8). When the concentrations of the cyclic peptides were increased to 21.2 or 42.4 μM, the fluorescence intensity was higher than the intensity of 10.6 μM, indicating that the membrane and cellular integrity could be changed. The results reveal that cyclic peptide-1, -11, and -14 had no effect on membrane permeability, and this caused intracellular DNA and other intracellular material leakage, accelerating the death of bacteria.

### 2.8. Combinational Activities against MRSA

To verify further the impact on membrane permeability, we measured the combinational activities of cyclic peptides with ciprofloxacin and linezolid against MRSA ATCC43300. The outcomes of the cyclic peptide combinations with ciprofloxacin and linezolid against MRSA ATCC43300 are shown in Table 3. The FICI values show that the combinations had indifferent activities against MRSA ATCC43300, indicating that cyclic peptide-1, -11, and -14 had no impact on membrane permeability.

## 3. Discussion

We investigated the susceptibility of MRSA to cyclic peptide-1, -11, and -14. Cyclic peptide-1, -11, and -14 have a strong effect on antibiotic activity against planktonic MRSA. We also measured the antimicrobial activity of cyclic peptide-1, -11, and -14 against ESKAPE pathogens, and the results indicated that cyclic peptide-1, -11, and -14 are narrow-spectrum antimicrobial peptides. Next, cyclic peptide-1, -11, and -14 were evaluated for their potency as therapeutic agents. The MBC values of cyclic peptide-1, -11, and -14 were 4–8 times higher than the MIC values, suggesting that cyclic peptide-1, -11, and -14 exert strong bacteriostatic activities. Time-kill studies were conducted to evaluate the antimicrobial activity of cyclic peptide-1, -11, and -14 against MRSA over 24 h. The results indicate that cyclic peptide-1, -11, and -14 are critical for antibacterial activity against MRSA.

Bacterial biofilms are difficult to eradicate due to highly resilient microbial assemblies [35,36,37]. Unfortunately, drugs on anti-biofilm are rarely reported, making biofilm-caused infections extremely difficult to treat. In clinical settings, vancomycin, as the last line of defense against MRSA, is highly sensitive to MRSA infection, but it is not effective in dispersing MRSA biofilms [38]. Biofilms can prevent antibiotics from entering, protecting the bacteria inside from being killed [39,40]. Resistance caused by biofilms impedes the treatment of many chronic MRSA biofilm-related infections, including osteomyelitis, endocarditis, and indwelling medical device infections [41,42]. Recent studies have highlighted that AMPs might prevent biofilm formation or be used to treat established biofilms [28]. In our study, cyclic peptide-1, -11, and -14 were demonstrated to have poor inhibition activity towards biofilm formation by MRSA43390. They also have no obvious inhibition activity towards other bacterial biofilms, such as *Pseudomonas aeruginosa*. Surprisingly, they showed an ability to disperse bacterial biofilms. Although some antibiotics have been show to enter the biofilm matrix, they have no antibacterial activity against the dormant subpopulation of persister cells [43]. Given the efficacies of cyclic peptides in dispersing biofilms, we assessed their ability to kill MRSA persister cells that can be generated in the laboratory by treating exponentially growing bacteria with a high concentration of ciprofloxacin. Together, the effects of cyclic peptide-1, -11, and -14 on the biofilm and persisters make it a promising antibiofilm and anti-persister agent.

The development of resistance is a great challenge that hampers drug development and the eradication of nosocomial pathogens [44,45]. To determine whether MRSA is susceptible to developing resistance to these cyclic peptides, we demonstrated the lack of the development of resistance to cyclic peptide-1, -11, and -14 in MRSA when cultured for 20 passages in the presence of subinhibitory cyclic peptide concentrations. This lack of development supported the potential for further clinical development. In addition, as antibacterial agents must have strong antimicrobial activity and combine with low cytotoxicity against mammalian cells, we assessed the hemolytic activity of cyclic peptides towards red blood cells and the cytotoxicity against human liver L02 cells. Our results reveal that cyclic peptide-1, -11, and -14 are safe, promising candidate compounds.

Some antibiotics, in order to kill the bacteria, must enter the bacterial cell to target the bacterial DNA helicase or protein synthesis. However, bacteria such as MRSA produce a bacterial membrane that reduces the effectiveness of antibiotics against bacteria. By increasing the permeability of the bacterial membrane, the activity of antibiotics may be improved. Therefore, we further investigated the combinational activities of cyclic peptides with ciprofloxacin and linezolid against MRSA. If cyclic peptide-1, -11, and -14 have an impact on membrane permeability, then the MIC of the combination should be diminished. Our results demonstrated that neither cyclic peptide-1, -11, nor -14 caused any change of membrane permeability. The basic mechanisms at work in killing might be different from those at work in growth inhibition. Peptide localization in cells will be further investigated, and this may provide more evidence for the mechanisms of action of cyclic peptide-1, -11, and -14.

## 4. Materials and Methods

### 4.1. Cyclic Peptide-1, Cyclic Peptide-11, and Cyclic Peptide-14

Cyclic peptide-1 and the analogues cyclic peptide-11 and cyclic peptide-14 in the study, summarized in Table 4, were synthesized with procedures reported [32,33]. Briefly, the total chemical synthesis of cyclic peptide-1 was achieved by a strategy-based, manual, solid-phase peptide synthesis. Amino acids were coupled. Valine positions were coupled twice. Deprotection was performed in trifluoroacetic acid for 30 min. Peptides were cleaved from the resin with acetonitrile–water–trifluoroacetic acid (79.95:20:0.05) for 30 min. Lyophilisation yielded the crude product. The crude synthetic lugdunin product was purified by reversed-phase HPLC and compared with the natural product by electrospray ionization liquid chromatography high-resolution mass spectrometry, additional chiral-HPLC methods. The analogues, cyclic peptide-11 and cyclic peptide-14, were synthesized by replacing some pharmacophore positions of cyclic peptide-1. All compounds were dissolved in dimethylsulfoxide and stock solutions and stored at −80 °C.

### 4.2. Bacterial Strains and Growth Media

Media used were tryptic soy broth supplemented with 0.5% glucose for biofilm production, cation-adjusted Mueller-Hinton broth for the minimal inhibitory concentration (MIC), and tryptic soy agar for the minimum bactericidal concentration (MBC). Luria broth was used for in vitro culture. All media are from Solarbio, Beijing China. The MRSA strains used in this study were MRSA43300, MRSA3390, MRSA3392, MRSA3394, MRSA3397, and MRSA3398. Other strains include *Enterococcus faecium*, *Klebsiella pneumoniae*, *Acinetobacter baumannii*, *Pseudomonas aeruginosa*, and *Enterobacter* sp. Bacterial strains were stored in a refrigerator at −80 °C.

### 4.3. MIC and MBC Determinations

MIC assays were adapted according to the recommendations of the Clinical and Laboratory Standard Institute (CLSIM) [46,47]. MBCs were determined at the end of the incubation period by removing 20 µL samples from wells showing no growth on MHA plates, which were incubated for 24 h and examined for 99.9% killing.

### 4.4. Time-Kill Curves

A stationary-phase culture of MRSA ATCC43300 was diluted to 10^5^ CFU/mL in a growth medium. The final concentrations of the cyclic peptides in the bacterial suspension were adjusted to 5.3, 21.2, 42.4, and 84.8 μM following the time-kill methodology [48]. The final concentration of vancomycin in the bacterial suspension was adjusted to 1.4 μM as a postive control. At 4, 8, 16, 24, and 30 h, samples were centrifuged. The pellet was resuspended in PBS and serially diluted. Aliquots (10 μL) were then spread on agar plates, and colonies were counted after a 24 h culture at 37 °C. Bactericidal activity (99.9% kill) was defined as a ΔLog_10_CFU/mL ≥3 at 24 h in a colony count from the initial inoculum. Bacteriostatic activity was defined as a ΔLog_10_CFU/mL <3 at 24 h in a colony count from the initial inoculum.

### 4.5. Inhibition of Biofilm Formation

The inhibition of biofilm formation was assessed as described previously [49]. Briefly, a stationary-phase culture of MRSA 3390 (1 × 10^6^ CFU/mL) in TSB-0.5% glucose was incubated in 24-well tissue culture microtiter plates at 37 °C for 24 h, with cyclic peptide-1, -11, and -14 at 0 µM, 2.6 µM, and 5.3 µM in 96-well tissue culture microtiter plates. The positive control was MRSA 3390 in TSB-0.5% glucose without cyclic peptides. After incubation, the wells were decanted and washed twice with phosphate buffer saline (pH 7.3). The wells were then stained with crystal violet (0.4%). The OD_595_ was measured.

### 4.6. Biofilm Dispersal

A stationary-phase culture of MRSA 3390 (1 × 10^6^ CFU/mL) in TSB-0.5% glucose was incubated at 37 °C for 48 h. The wells were washed with phosphate buffer saline (pH 7.3) three times. The biofilm in the phosphate buffer saline (PBS) was incubated at 37 °C for 24 h with cyclic peptide-1, cyclic peptide-11, and cyclic peptide-14 at 2.6 µM, 5.3 µM, 10.6 µM, and 21.2 µM in 24-well tissue culture microtiter plates. As a control, biofilms were exposed to PBS without cyclic peptide. After incubation, the wells were decanted and washed three times with phosphate buffer saline (pH 7.3). The wells were then stained with crystal violet (0.4%) [50]. The OD_595_ was measured. Results were interpreted by a comparison of cyclic peptides on treated biofilms with those on untreated biofilms.

### 4.7. Antibacterial Properties against Persister Cells

MRSA ATCC43300 persister cells were generated by diluting a stationary phase culture of MRSA ATCC43300 bacteria at 1:1000 in an LB medium and grown to OD_600_ 0.5 at 37 °C with 200 rpm shaking. The bacteria cells were collected by centrifugation (5000 rpm, 5 min, 4 °C) and washed with sterile PBS. The bacteria were resuspended in PBS to a density of OD_600_ 0.5. The OD_600_ 0.5 culture was treated with 20 × MIC of ciprofloxacin (8 μg/mL treatment) for 6 h at 37 °C with 200 rpm shaking. The bacteria resuspension was treated with different concentrations of cyclic peptide-1, -11, and -14. The number of colonies was counted after 24 h of culture at 37 °C. Ciprofloxacin (final concentration 8 μg/mL, 20 × MIC) and DMSO (0.1%, *v*/*v*) were used as controls.

### 4.8. Antibacterial Activities of Combinations against MRSA

Combinational activities of cyclic peptide-1, -11, and -14 with linezolid and ciprofloxacin against MRSAATCC43300 were determined by the modified broth microdilution checkerboard technique [51]. MRSA ATCC43300 was cultured to an exponential phase and diluted to 1 × 10^5^ CFU/mL with fresh culture broth. The lowest treatment concentration where no bacterial growth occurred was regarded as the MIC, which was determined by OD_600_ measurements on a microplate reader. The fractional inhibitory concentration index (FICI) was determined as the inhibitory concentration of the combination divided by that of the single antibiotic. The combination index was derived from the highest dilution of antibiotic combination permitting no visible growth. With this method, synergy was defined as an FICI of <0.5, no interaction was defined as an FICI of >0.5 to 4, and antagonism was defined as an FICI of >4.0.

### 4.9. Confocal Microscopy of Biofilms

MRSA3390 was grown in TSB shaken at 37 °C until the stationary phase was reached. The cultures were diluted to a final inoculum density of 1 × 10^5^ CFU/mL. A 900 μL portion of inoculum was added to each well with a coverglass slide, and the biofilms were grown statically in a humidified chamber at 37 °C for 48 h [50]. The biofilms were incubated with cyclic peptide-1, -11, and -14 statically for 24 h at 37 °C. The coverslips were removed from the wells, rinsed with sterile water to remove any planktonic cells, and dried at 50 °C for 20 min. A solution of live/dead bacterial stain, BacLight (Invitrogen, Waltham, MA, USA), was used to stain the biofilm and imaged using a Leica TCS SP8 confocal microscope.

### 4.10. Membrane Permeability Assay

MRSA ATCC43300 was grown overnight in LB at 37 °C. The cells were incubated in fresh LB to 3.5 × 10^8^ CFU/mL. The bacterial cells were harvested by centrifugation at 5000 rpm for 5 min and washed with phosphate buffer saline thrice. The bacterial cells were resuspended in phosphate buffer saline to a final concentration of 10^8^ CFU/mL. The cell suspension (50 μL) was incubated with 50 μL of peptide solutions at various concentrations (1.3–84.8 μM) in phosphate buffer for 60 min at 37 °C [52]. Afterwards, 5 μL of propodium iodide (PI) at 1 µM was added to the cell suspension, and measurements were carried out in black polystyrene microtiter plates using a fluorimeter equipped with 535 nm excitation and 615 nm emission filters.

### 4.11. Resistance Evolution Experiment

To evaluate the propensity of cyclic peptide-1, -11, and -14 to induce resistance, an MIC assay was performed for 20 days using MRSA ATCC43300, where each assay was incubated 24 h at 37 °C. Bacteria from the 1/2 MIC well the prior day were used to generate the inoculum for that day’s assay.

### 4.12. Hemolytic Assay

Sheep red blood cells were diluted in PBS to 5%. In each well of a clear, flat-bottom 96-well plate, 100 µL of sheep red blood cells were placed, followed by an addition of 100 µL of a cyclic peptide solution, to a final peptide concentration of 10.6–84.8 µM. As a positive control, 1% Triton X-100 was used. As a negative control, 100 µL of PBS was used. Plates were incubated for 1 h at 37 °C. The plates were centrifuged for 10 min at 1000× *g*. Supernatants (150 µL) were transferred to a new plate, and absorbance at 540 nm was measured. The degree of hemolysis of the cyclic peptides was expressed in percent relative to the hemolysis caused by 1%Triton-X [34].

### 4.13. Cytotoxicity

Cytotoxicity was determined using mammalian cells. Briefly, L02 cells (5000 cells/well) were cultured in 96-well plates with Dulbecco’s modified Eagle’s medium containing 10% FBS and penicillin (100 U/mL)-streptomycin (100 µg/mL) at 37 °C in a humidified 5% CO_2_ atmosphere. After 24 h of incubation, fresh media with or without the testing peptides at different concentrations were added to the wells and incubated for another 24 h under the same conditions. Cell viability was determined by the reduction of a tetrazolium compound by viable cells, generating a colored formazan dye soluble in cell culture media. After 24 h of incubation with testing samples, a fresh medium and 20 µL of MTT (5 mg/mL) was added to each well and incubated for 4 h in standard cell growth conditions, and the absorbance at 490 nm of the resulting solution was measured [53].

### 4.14. Statistical Analysis

All assays were carried out in triplicate, and the results were calculated and compared with those of the control groups. All statistical data were analyzed by GraphPad Prism statistical software. *p* values of <0.05 were assumed to be statistically significant.

## 5. Conclusions

In this study, we showed that cyclic peptide-1, -11, and -14 exhibit novel activities against MRSA populations that are difficult to treat and counteract clinically relevant MRSA biofilms, which are of high relevance due to their relation with recurrent skin and soft tissue infections. Cyclic peptide-1, -11, and -14 possess great dispersion biofilm properties against MRSA and kill MRSA persister cells with non-toxicity. They lower hemolytic activity and lack resistance development. Thus, we show here that cyclic peptide-1, -11, and -14 are excellent drug candidates for combating MRSA infections.

## Figures and Tables

**Table 1:** MIC and MBC tests of cyclic peptides against different MRSA bacteria.

MIC and MBC Test of Cyclic Peptide against Different MRSA Bacteria								
Compound	Value	MRSA 43300	MRSA 3390	MRSA 3392	MRSA 3394	MRSA 3396	MRSA 3397	MRSA 3398
cyclic peptide-1	MIC (μM)	5.1	5.1	5.1	5.1	5.1	5.1	5.1
	MBC (μM)	81.5	20.4	40.8	20.4	20.4	20.4	20.4
cyclic peptide-11	MIC (μM)	10.6	10.6	10.6	10.6	10.6	10.6	10.6
	MBC (μM)	84.8	84.8	84.8	42.4	42.4	42.4	21.2
cyclic peptide-14	MIC (μM)	10.6	10.6	10.6	10.6	10.6	10.6	10.6
	MBC (μM)	84.8	42.4	84.8	84.8	84.8	42.4	84.8
vancomycin	MIC (μM)	1.4	0.69	0.69	1.4	1.4	1.4	1.4
	MBC (μM)	2.8	1.4	1.4	2.8	2.8	2.8	2.8

**Table 2:** MICs (μM) of cyclic peptides against the ESKAPE pathogen.

MIC (μM) of Cyclic Peptides against ESKAPE Pathogen				
	Compound	Cyclic Peptide-1	Cyclic Peptide-11	Cyclic Peptide-14
Strains				
*Enterococcus faecium*		>163	>169.5	>169.5
*Klebsiella pneumoniae*		>163	>169.5	>169.5
*Acinetobacter baumannii*		>163	>169.5	>169.5
*Pseudomonas aeruginosa*		>163	>169.5	>169.5
*Enterobacter* sp.		>163	>169.5	>169.5

**Table 3:** The activities of antibiotics in combination with antibiotics against MRSA ATCC 43300.

Antibiotic Combinations	FICI Ratio
cyclic peptide-1–ciprofloxacin	1
cyclic peptide-1–linezolid	3
cyclic peptide-11–ciprofloxacin	1
cyclic peptide-11–linezolid	3
cyclic peptide-14–ciprofloxacin	1
cyclic peptide-14–linezolid	4

**Table 4:** Cyclic peptides used in the study.

Compound	Structure
cyclic peptide-1	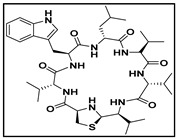
cyclic peptide-11	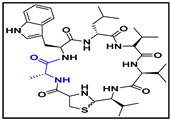
cyclic peptide-14	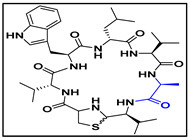

## Data Availability

The data are contained within this publication.

## Abbreviations

MIC	minimal inhibitory concentration
MRS	methicillin-resistant *Staphylococcus aureus*
EPS	extracellular polysaccharide
MTT	methyl thiazolyltetrazolium
PI	propidium iodide
CFU	colony forming unit

## References

[1] Ramanan L., Precious M., Suraj P., Charles B., John-Arne R., Keith K., Sally D. (2016). Access to effective antimicrobials: A worldwide challenge. Lancet.

[2] Walsh T.R. (2021). A one-health approach to antimicrobial resistance. Nat. Microbiol..

[3] Oliveira D., Forde B.M., Kidd T.J. (2020). Antimicrobial resistance in ESKAPE pathogens. Clin. Microbiol. Rev..

[4] Willyard C. (2017). The drug-resistant bacteria that pose the greatest health threats. Nature.

[5] Potera C., Han J., Craighead H.G. (1999). Forging a link between biofilms and disease. Science.

[6] Simões M., Bennett R.N., Rosa E. (2009). Understanding antimicrobial activities of phytochemicals against multidrug resistant bacteria and biofilms. Nat. Prod. Rep..

[7] Evans J.J., Bolz D.D. (2019). Regulation of virulence and antibiotic resistance in Gram-positive microbes in response to cell wall-active antibiotics. Curr. Opin. Infect. Dis..

[8] Flemming H.C., Wingender J., Szewzyk U., Steinberg P., Rice S.A., Kjelleberg S. (2016). Biofilms: An emergent form of bacterial life. Nat. Rev. Microbiol..

[9] Verderosa A.D., Totsika M., Fairfull-Smith K.E. (2019). Bacterial biofilm eradication agents: A current Review. Front. Chem..

[10] Flemming H.C., Wingender J. (2010). The biofilm matrix. Nat. Rev. Microbiol..

[11] Sharma D., Misba L., Khan A.U. (2019). Antibiotics versus biofilm: An emerging battleground in microbial communities. Antimicrob. Resist. Infect. Control.

[12] Kumar P., Lee J.H., Beyenal H. (2020). Fatty acids as antibiofilm and antivirulence agents. Trends Microbiol..

[13] Hughes G., Webber M.A. (2017). Novel approaches to the treatment of bacterial biofilm infections. Br. J. Pharmacol..

[14] Li C.H., Chen X., Landis R.F. (2019). Phytochemical-based nanocomposites for the treatment of b acterial biofilms. ACS Infect. Dis..

[15] Philip A., Stewart S., Costerton J.W. (2019). Antibiotic resistance of bacteria in biofilms. Lancet.

[16] Lebeaux D., Ghigo J.M., Beloin C. (2014). Biofilm-related infections: Bridging the gap between clinical management and fundamental aspects of recalcitrance toward antibiotics. Microbiol. Mol. Biol. Rev..

[17] Davies D. (2003). Understanding biofilm resistance to antibacterial agents. Nat. Rev. Drug Discov..

[18] Liu S., Brul S., Zaat S. (2020). Bacterial Persister-Cells and Spores in the Food Chain: Their potential inactivation by antimicrobial peptides (AMPs). Int. J. Mol. Sci..

[19] Pozo J., Patel R. (2007). The challenge of treating biofilm-associated bacterial infections. Clin. Pharmacol. Ther..

[20] Abebe G.M. (2020). The Role of Bacterial Biofilm in Antibiotic Resistance and Food Contamination. Int. J. Microbiol..

[21] Olsen I. (2015). Biofilm-specific antibiotic tolerance and resistance. Eur. J. Clin. Microbiol. Infect. Dis..

[22] Penesyan A., Gillings M., Paulsen I. (2015). Antibiotic discovery: Combatting bacterial resistancein cells and in biofilm communities. Molecules.

[23] Galdiero E., Lombardi L., Falanga A. (2019). Biofilms: Novel strategies based on antimicrobial peptides. Pharmaceutics.

[24] Wolfmeier H., Pletzer D., Mansour S.C., Hancock R. (2018). New perspectives in biofilm eradicati on. ACS Infect. Dis..

[25] Kang H.K., Kim C., Seo C.H., Park Y. (2017). The therapeutic applications of antimicrobial peptides (AMPs): Apatent review. J. Microbiol..

[26] Mishra B., Reiling S., Zarena D., Wang G. (2017). Host defense antimicrobial peptides as antibiotics: Design and application strategies. Curr. Opin. Chem. Biol..

[27] Pletzer D., Hancock W. (2016). Antibiofilm peptides: Potential as broad-spectrum agents. J. Bacteriol..

[28] Breij A.D., Riool M., Cordfunke R.A. (2013). The antimicrobial peptide SAAP-148 combats drug-resistant bacteria and biofilms. Sci. Transl. Med..

[29] Haney E.F., Hancock R.E. (2013). Peptide design for antimicrobial and immunomodulatory applications. Biopolymers.

[30] Saikia K., Sravani Y.D., Ramakrishnan V., Chaudhary N. (2017). Highly potent antimicrobial peptides from N-terminal membrane-binding region of *E. coli* MreB. Sci. Rep..

[31] Vishwakarma A., Dang F., Ferrell A. (2021). Peptidomimetic polyurethanes disrupt surface established bacterial biofilms and prevent biofilm formation. J. Am. Chem. Soc..

[32] Zipperer A., Konnerth M.C., Laux C. (2016). Human commensals producing a novel antibiotic impair pathogen colonization. Nature.

[33] Schilling N.A., Berscheid A., Schumacher J. (2009). Synthetic lugdunin analogues reveal essential structural motifs for antimicrobial action and proton translocation capability. Angew. Chem. Int. Ed..

[34] Ma Z., Han J., Chang B. (2017). Membrane-active amphipathic peptide wrl3 with in vitro antibio film capability and in vivo efficacy in treating methicillin-resistant staphylococcus aureus burn wound infections. ACS Infect. Dis..

[35] Gerdes K., Semsey S. (2016). Pumping persisters. Nat. Cell Biol..

[36] Thorn C.R., Howell P.L., Wozniak D.J., Prestidge C.A., Thomas N. (2021). Enhancing the therapeutic use of biofilm-dispersing enzymes with smart drug delivery systems. Adv. Drug Deliv. Rev..

[37] Roilides E., Simitsopoulou M., Katragkou A., Walsh T.J. (2015). How biofilms evade host defenses. Microbiol. Spectr..

[38] Wender P.A., Huttner M.A., Staveness D., Vargas J.R., Xu A.F. (2015). Guanidinium-rich, glycerol-derived oligocarbonates. A new class of cell-penetrating molecular transporters that complex, deliver, and release sirna. Mol. Pharm..

[39] Fux C.A., Wilson S., Stoodley P. (2004). Detachment characteristics and oxacillin resistance of staphyloccocus aureus biofilm emboli in an in vitro catheter infection model. J. Bacteriol..

[40] Yarwood J.M., Bartels D.J., Volper E.M., Greenberg E.P. (2004). Quorum sensing in staphylococcus aureus biofilms. J. Bacteriol..

[41] Costerton W., Veeh R., Shirtliff M., Pasmore M., Post C., Ehrlich G. (2003). The application of biofilm science to the study and control of chronic bacterial infections. J. Clin. Investig..

[42] Parsek M.R., Singh P.K. (2003). Bacterial biofilms: An emerging link to disease pathogenesis. Annu. Rev. Microbiol..

[43] Iris K., Niilo K., Amy S., Yipeng W., Kim L. (2004). Persister cells and tolerance to antimicrobials. FEMS Microbiol. Lett..

[44] Khan Z., Faisal S., Hasnain S. (2019). The Continuing Threat of Methicillin-Resistant Staphylococcus aureus. Antibiotics.

[45] Mermel L., Cartony J.M., Covington P., Maxey G., Morse D. (2011). Methicillin-Resistant Staphylococcus aureus colonization at different body sites: A prospective, quantitative analysis. J. Clin. Microbiol..

[46] Tenover F.C., Moellering R.C. (2007). The rationale for revising the clinical and laboratory standards institute vancomycin minimal inhibitory concentration interpretive criteria for s taphylococcus aureus. Clin. Infect. Dis..

[47] Horn K.S.V., Burda W.N., Fleeman R., Shaw L.N., Manetsch R. (2014). Antibacterial activity of a series of n2,n4-disubstituted quinazoline-2,4-diamines. J. Med. Chem..

[48] Eckert R., Qi F., Yarbrough D.K., He J., Anderson M.H., Shi W. (2006). Adding selectivity to antimicrobial peptides. Rational design of a multidomain peptide against *Pseudomonas* spp.. Antimicrob. Agents Chemother..

[49] Sambanthamoorthy K., Sloup R.E., Parashar V., Smith J.M., Kim E.E., Semmelhack M.F. (2012). Identification of small molecules that antagonize diguanylate cyclase enzymes to inhibit biofilm formation. Antimicrob. Agents Chemother..

[50] Yasir M., Dutta D., Willcox M.D.P. (2020). Activity of antimicrobial peptides and ciprofloxacin against pseudomonas aeruginosa biofilms. Molecules.

[51] Mataraci E., Dosler S. (2012). In vitro activities of antibiotics and antimicrobial cationic peptides alone and in combination against methicillin-resistant staphylococcus aureus biofilms. Antimicrob. Agents Chemother..

[52] De Zoysa G.H., Cameron A.J., Hegde V.V., Raghothama S., Sarojini V. (2015). Antimicrobial peptides with potential for biofilm eradication: Synthesis and structure activity relationship studies of battacin peptides. J. Med. Chem..

[53] Jayathilaka E., Rajapaksha D.C., Nikapitiya C. (2021). Antimicrobial and Anti-Biofilm Peptide Octominin for Controlling Multidrug-Resistant Acinetobacter baumannii. Int. J. Mol. Sci..

